# Supplementary material for: Collective dynamics of stock market efficiency
Source: Sci Rep. 2020 Dec 15;10:21992. doi: 10.1038/s41598-020-78707-2 (PMC7738547; doi:10.1038/s41598-020-78707-2)
Supplement: Supplementary file 1 — Supplementary material 1 [file 41598_2020_78707_MOESM1_ESM.pdf]

Supplementary Information for

# Collective dynamics of stock market efficiency

Luiz G. A. Alves, Higor Y. D. Sigaki, Matjaž Perc, and Haroldo V. Ribeiro

Scientific Reports, 2020

| | Stock market index | Location | Ticker symbol | Data source |
|---|---|---|---|---|
| 1 | MERVAL | Argentina | ^MERV | Yahoo finance |
| 2 | ALL ORDINARIES | Australia | ^AORD | Yahoo finance |
| 3 | S&P/ASX 200 | Australia | ^AXJO | Yahoo finance |
| 4 | ATX Index | Austria | ^ATX | Yahoo finance |
| 5 | BEL 20 | Belgium | ^BFX | Yahoo finance |
| 6 | IBOVESPA | Brazil | ^BVSP | Yahoo finance |
| 7 | S&P/TSX Composite index | Canada | ^GSPTSE | Yahoo finance |
| 8 | SSE Composite Index | China | 000001.SS | Yahoo finance |
| 9 | Shenzhen Component | China | 399001.SZ | Yahoo finance |
| 10 | EURONEXT 100 | Europe | ^N100 | Yahoo finance |
| 11 | STOXX Europe 50 Index | Europe | ^STOXX50E | Wall Street Journal |
| 12 | CAC 40 | France | ^FCHI | Yahoo finance |
| 13 | DAX PERFORMANCE-INDEX | Germany | ^GDAXI | Yahoo finance |
| 14 | HANG SENG INDEX | Hong Kong | ^HSI | Yahoo finance |
| 15 | S&P BSE SENSEX | India | ^BSESN | Yahoo finance |
| 16 | Jakarta Composite Index | Indonesia | ^JKSE | Yahoo finance |
| 17 | TA-35 Index | Israel | TA35.TA | Yahoo finance |
| 18 | FTSE Italia All-Share Index | Italy | FTSEMIB.MI | Yahoo finance |
| 19 | Nikkei 225 | Japan | ^N225 | Yahoo finance |
| 20 | FTSE Bursa Malaysia KLCI | Malaysia | ^KLSE | Yahoo finance |
| 21 | IPC MEXICO | Mexico | ^MXX | Yahoo finance |
| 22 | Amsterdam AEX Index | Netherlands | ^AEX | Yahoo finance |
| 23 | Oslo Bors All Share Index | Norway | ^OSEAX | Wall Street Journal |
| 24 | Pakistan Stock Exchange | Pakistan | ^KSE | Yahoo finance |
| 25 | PSEi Index | Philippines | PSEI.PS | Yahoo finance |
| 26 | WIG20 | Poland | WIG20 | investing.com |
| 27 | MOEX Russia Index | Russia | IMOEX.ME | investing.com |
| 28 | Russian Trading System (RTS) Index | Russia | RTSI.ME | Wall Street Journal |
| 29 | Tadawul All Shares Index | Saudi Arabia | ^TASI.SR | investing.com |
| 30 | STI Index | Singapore | ^STI | Yahoo finance |
| 31 | Top 40 USD Net TRI Index | South Africa | ^JN0U.JO | Wall Street Journal |
| 32 | KOSPI Composite Index | South Korea | ^KS11 | Yahoo finance |
| 33 | IBEX 35 Index | Spain | ^IBEX | Yahoo finance |
| 34 | Swiss Market Index | Switzerland | ^SSMI | Yahoo finance |
| 35 | TSEC weighted index | Taiwan | ^TWII | Yahoo finance |
| 36 | SET Index | Thailand | ^SET.BK | investing.com |
| 37 | FTSE 100 | UK | ^FTSE | Yahoo finance |
| 38 | Dow 30 | USA | ^DJI | Yahoo finance |
| 39 | NYSE AMEX COMPOSITE INDEX | USA | ^XAX | Yahoo finance |
| 40 | NYSE COMPOSITE (DJ) | USA | ^NYA | Yahoo finance |
| 41 | Nasdaq | USA | ^IXIC | Yahoo finance |
| 42 | Russell 2000 | USA | ^RUT | Yahoo finance |
| 43 | S&P 500 | USA | ^GSPC | Yahoo finance |

**Table S1.** The 43 major world stock markets used in our study.

| Model | Abbreviation | Minimal description length |
|---|---|---|
| Stochastic block model | SBM | 55 |
| Nested degree corrected stochastic block model | Nested DCSBM | 48 |
| Degree corrected stochastic block model | DCSBM | 41 |
| Nested stochastic block model | Nested SBM | 29 |

**Table S2.** Model selection based on the minimal description length.

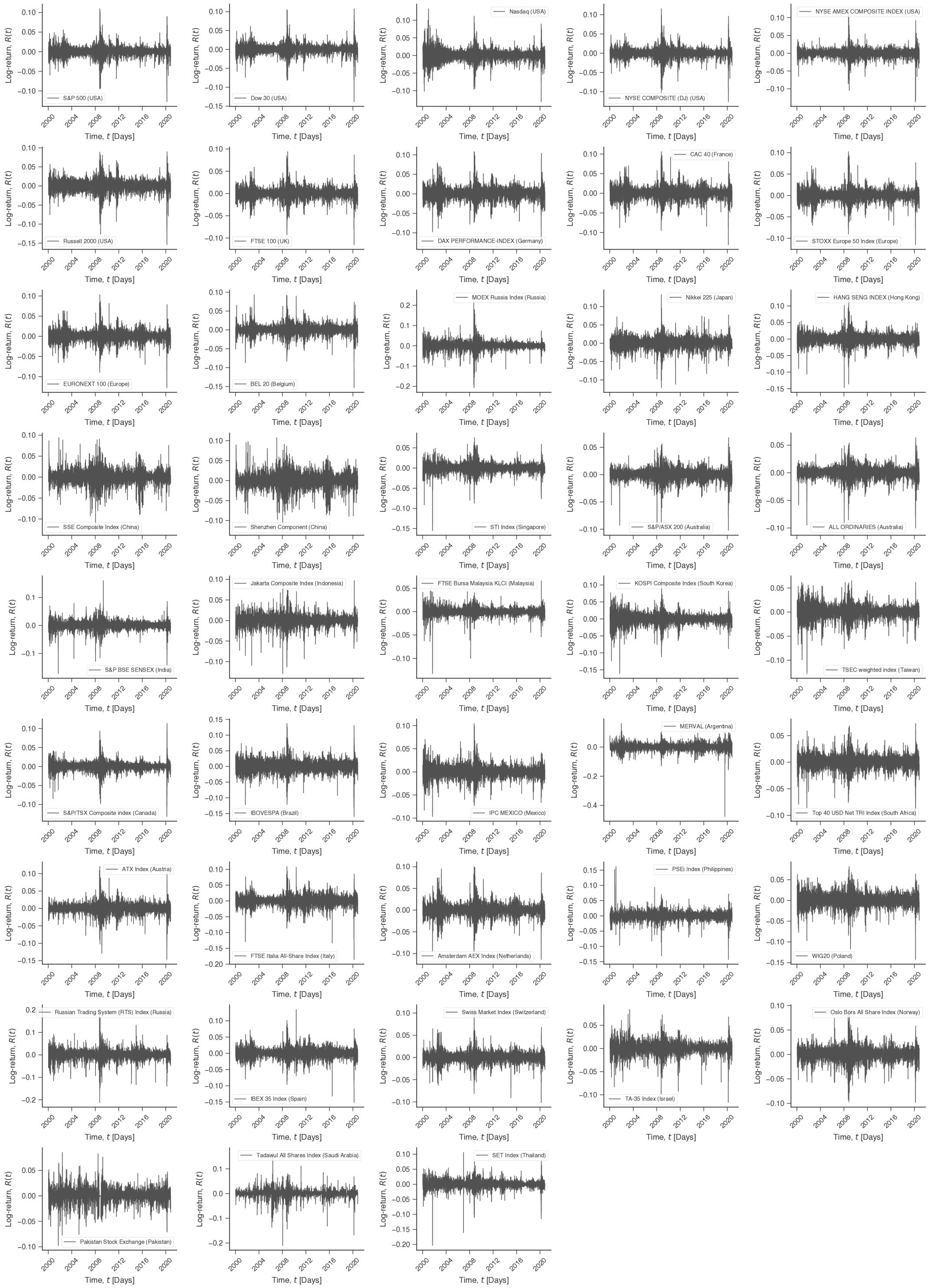


**Figure S1.** Log-returns $R(t)$ time series of the closing prices of the 43 world major stock markets indices from January 1, 2000 to October 31, 2020.

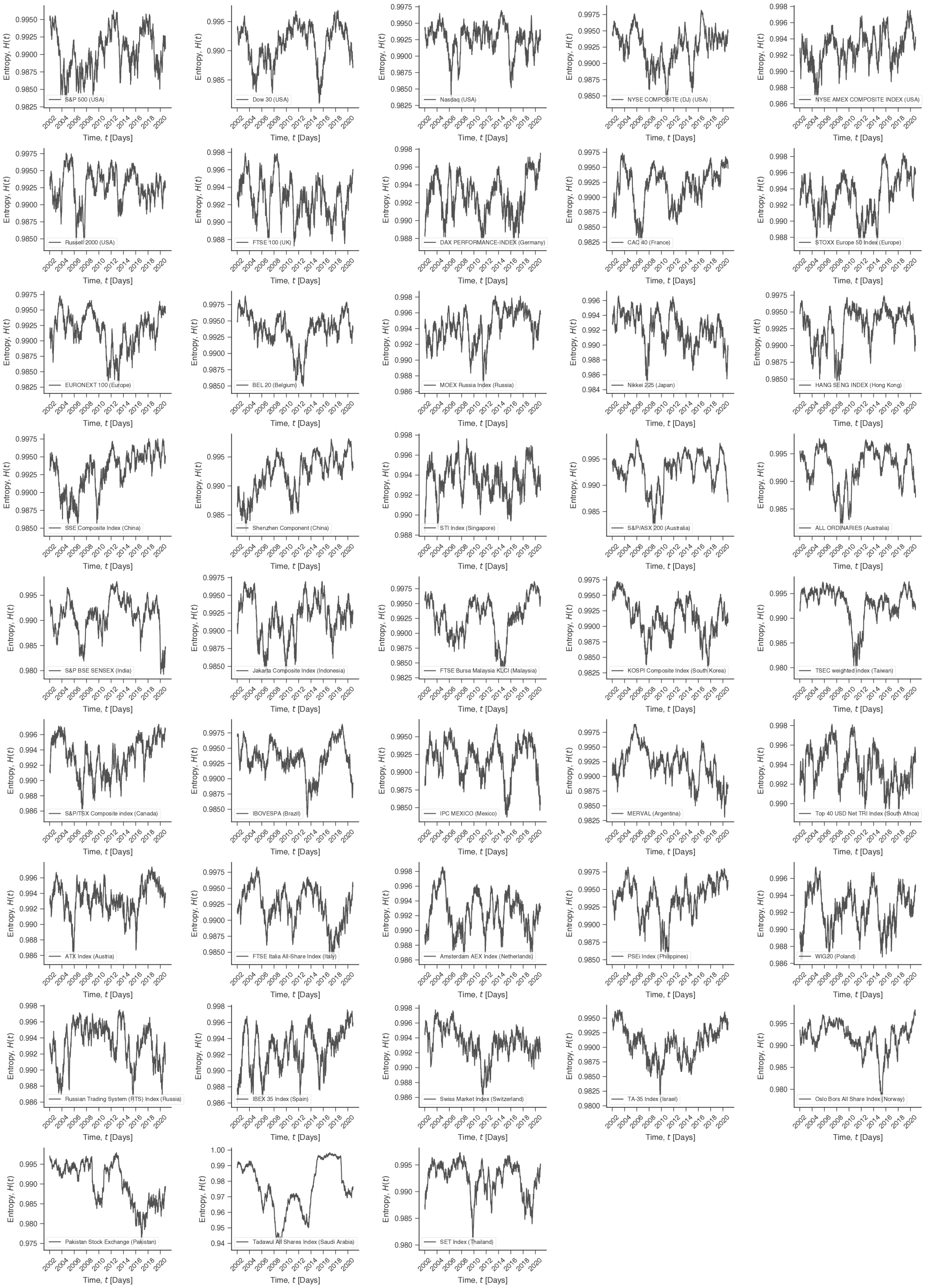


**Figure S2.** Time evolution of the permutation entropy $H(t)$ with embedding dimension $d = 4$ (see Methods Section for details) for the 43 major stock markets in our study.

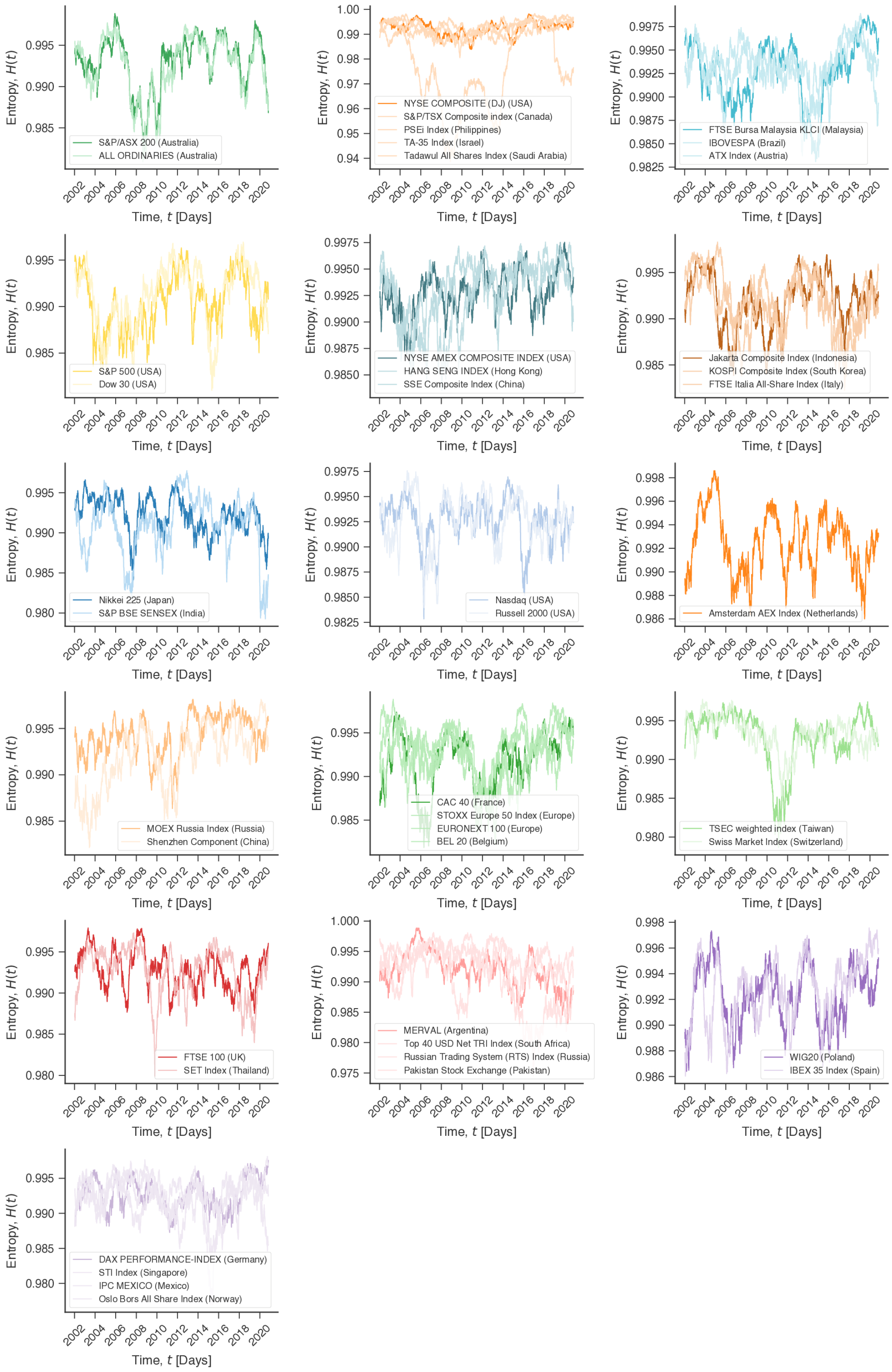


**Figure S3.** Time evolution of the entropy $H(t)$ for the 43 stock markets grouped according to the clusters obtained from the long-term dynamics of $H(t)$.

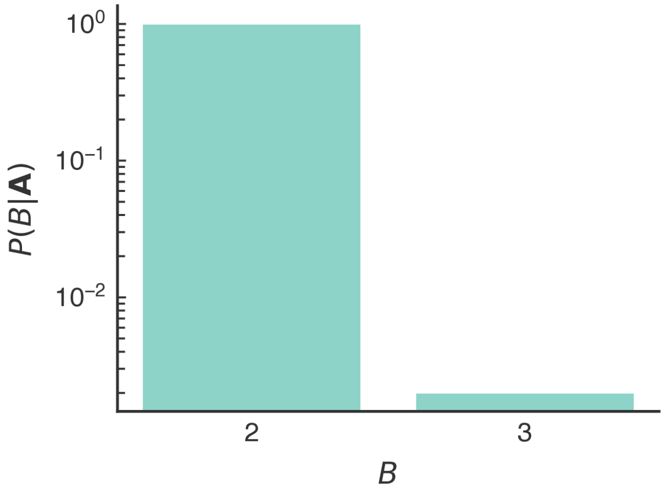


**Figure S4.** Marginal probability of group partition $P(B|\mathbf{A})$ in our financial network. The bar plot shows the probability density function $P(B|\mathbf{A})$ that the network $\mathbf{A}$ is partitioned into $B$ modules. The distribution is concentrated at $B = 2$, indicating that two modules is the most likely modular structure for our network. The probabilities were obtained by collecting the number of partitions for 10,000 sweeps of a Metropolis-Hastings acceptance-rejection Markov Chain Monte Carlo with multiple moves to sample hierarchical network partitions, at intervals of 10 sweeps.

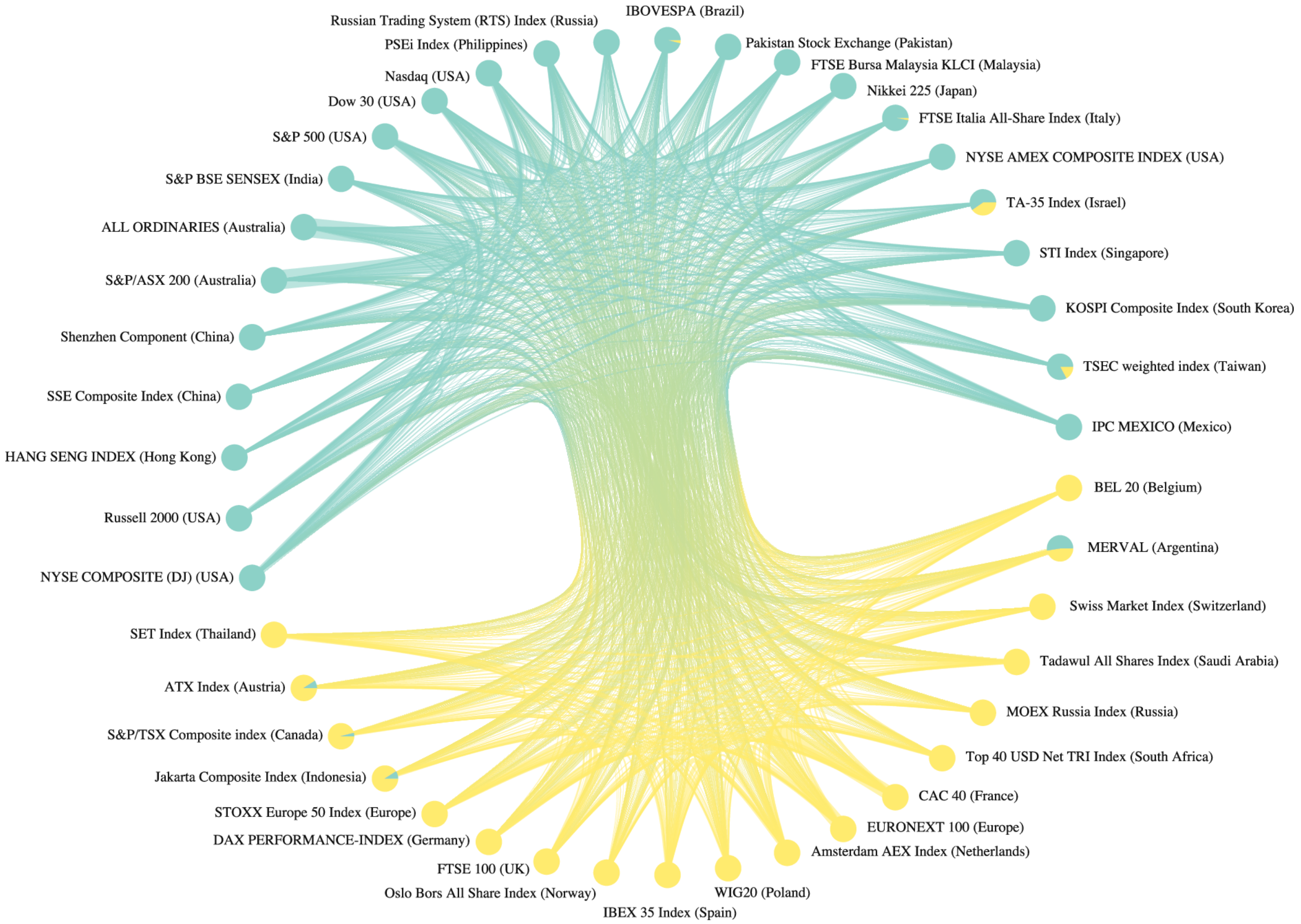


**Figure S5.** Marginal probabilities of node membership in our financial network. In this representation, nodes represent stock markets, and the pie divisions represent the marginal posterior probability that a node belongs to a given group (the two different colors). The probabilities were obtained by collecting the node membership for 10,000 sweeps of a Metropolis-Hastings acceptance-rejection Markov Chain Monte Carlo with multiple moves to sample hierarchical network partitions, at intervals of 10 sweeps. The edges and their weights have the same meaning as those from the network of Fig. 4 of the main text.
